# Right Heart Mass in Transit with a Hemorrhagic Pericardial Effusion: A Diagnostic Dilemma

**DOI:** 10.7759/cureus.4009

**Published:** 2019-02-04

**Authors:** Amit Nanda, Rami N Khouzam, John Jefferies, Marc Moon, Majesh Makan

**Affiliations:** 1 Internal Medicine, University of Tennessee, Memphis, USA; 2 Cardiology, University of Tennessee, Memphis, USA; 3 Surgery, Washington University, Saint Louis, USA; 4 Cardiology, Washington University, Saint Louis, USA

**Keywords:** cardiac imaging, cardiac metastasis

## Abstract

Metastatic disease to the heart is more often a post-mortem diagnosis due to non-specific symptoms and a low index of suspicion. Our case is a unique presentation of a rare case of cardiac metastasis from oropharyngeal cancer, which eluded echocardiographic diagnosis despite the presence of a hemorrhagic pericardial effusion. The cardiac metastasis, in fact, starts as pericardial seeding, as illustrated by the positron emission tomography (PET) imaging. The pericardial metastatic disease then becomes rapidly invasive into the cardiac chambers, hence presenting as a large mass on the echocardiogram and computed tomography (CT) scan of the chest. This is the first such case of pericardial metastasis from a squamous cell carcinoma of the tongue being reported and highlights the importance of an aggressive multimodality diagnostic approach in cases where such a clinical suspicion exists. While a two-dimensional (2D) echocardiogram is the most readily available modality, we recommend that this is complemented by the use of a three-dimensional (3D) echocardiogram, as well as metabolic and radiologic imaging with PET and CT scans.

## Introduction

In symptomatic patients, transthoracic echocardiography (TTE) and transesophageal echocardiography (TEE) can assess multiple cardiac diseases. Pericardial effusion can be quantified and assessment can be made for cardiac tamponade. Echocardiography can also guide pericardiocentesis, if needed. Intracardiac or pericardial masses may be identified and their impact on cardiac function can be evaluated using these modalities. The most important pitfall of TTE and TEE is the limited tissue characterization. We present the case of a patient with nonspecific acute symptoms and persistent hypotension with hemorrhagic pericardial effusion and non-resolving thrombus in transit. This was a rare and unique case of cardiac metastasis from a squamous cell carcinoma of the tongue that had escaped confirmatory diagnosis on repeated TTE and TEE until after an advanced disease process was identified on other non- echocardiographic imaging. This case highlights the importance of a multimodality diagnostic approach in such rare and difficult cases and the use of multimodality imaging to guide further workup and management. To date, no studies, trials, or guidelines exist delineating the superiority of any of these modalities.

## Case presentation

A 47-year-old white male presented with recurrent severe dizziness, chest tightness, shortness of breath, left upper back pain, night sweats, and chills of three weeks duration. His past medical history consisted of a 20 pack/year history of smoking and squamous cell carcinoma of the tongue, status-post excision with subsequent radiation and chemotherapy a year and a half prior to his presentation. He also had recurrent disease with biopsy-proven metastatic disease in the cervical lymph nodes nine months after the initial diagnosis and multiple lung nodules on CT and PET scans six months thereafter. He was continued on immunotherapy with nivolumab with subsequent cycles planned.

He was admitted for workup of the near syncope. A CT of the chest on admission revealed a filling defect in the left brachiocephalic vein extending into the right atrium and a filling defect in the right ventricle believed to be a thrombus. Heparin anticoagulation was initiated for possible thrombus in transit and a suspected pulmonary embolus (PE). Ten hours later, the patient developed severe diaphoresis, associated with inspiratory chest pain and shortness of breath. An electrocardiogram (ECG) revealed diffuse ST elevation, and a stat TTE revealed a large pericardial effusion with tamponade physiology. Emergent pericardiocentesis was performed and 1,600 cc bloody pericardial fluid was drained; the pericardial drain was left in for 24 hours with no significant additional drainage. Cytology of the pericardial fluid was negative. The patient’s shortness of breath persisted at rest and was associated with a low-grade fever (100.2° F). He experienced repeated episodes of hypotension and tachycardia, requiring intermittent fluid resuscitation and pressor support.

**Figure 1:**
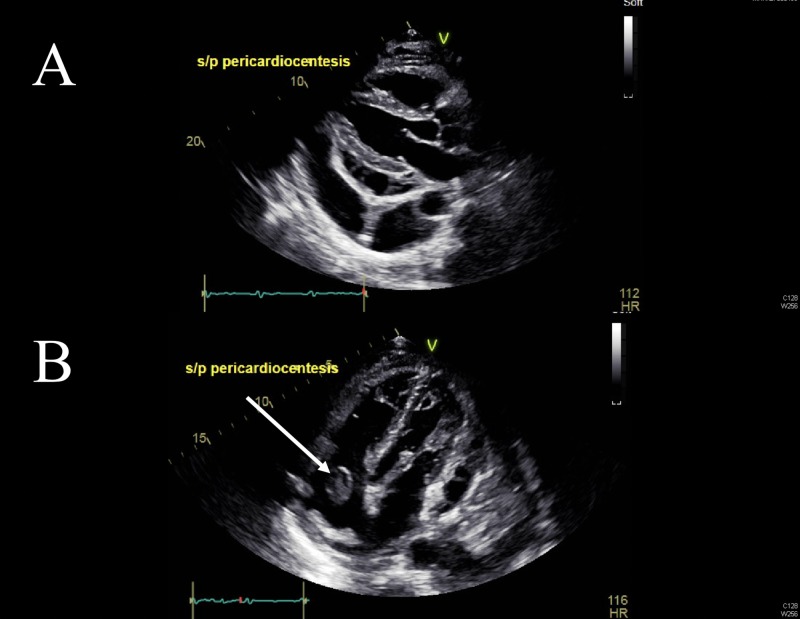
Presenting Transthoracic Echocardiographic Views A) Long axis and B) apical four chamber echocardiographic views at presentation showing the pericardial effusion and evidence of the thrombus in transition through the right atrium. S/P: status-post

**Figure 2:**
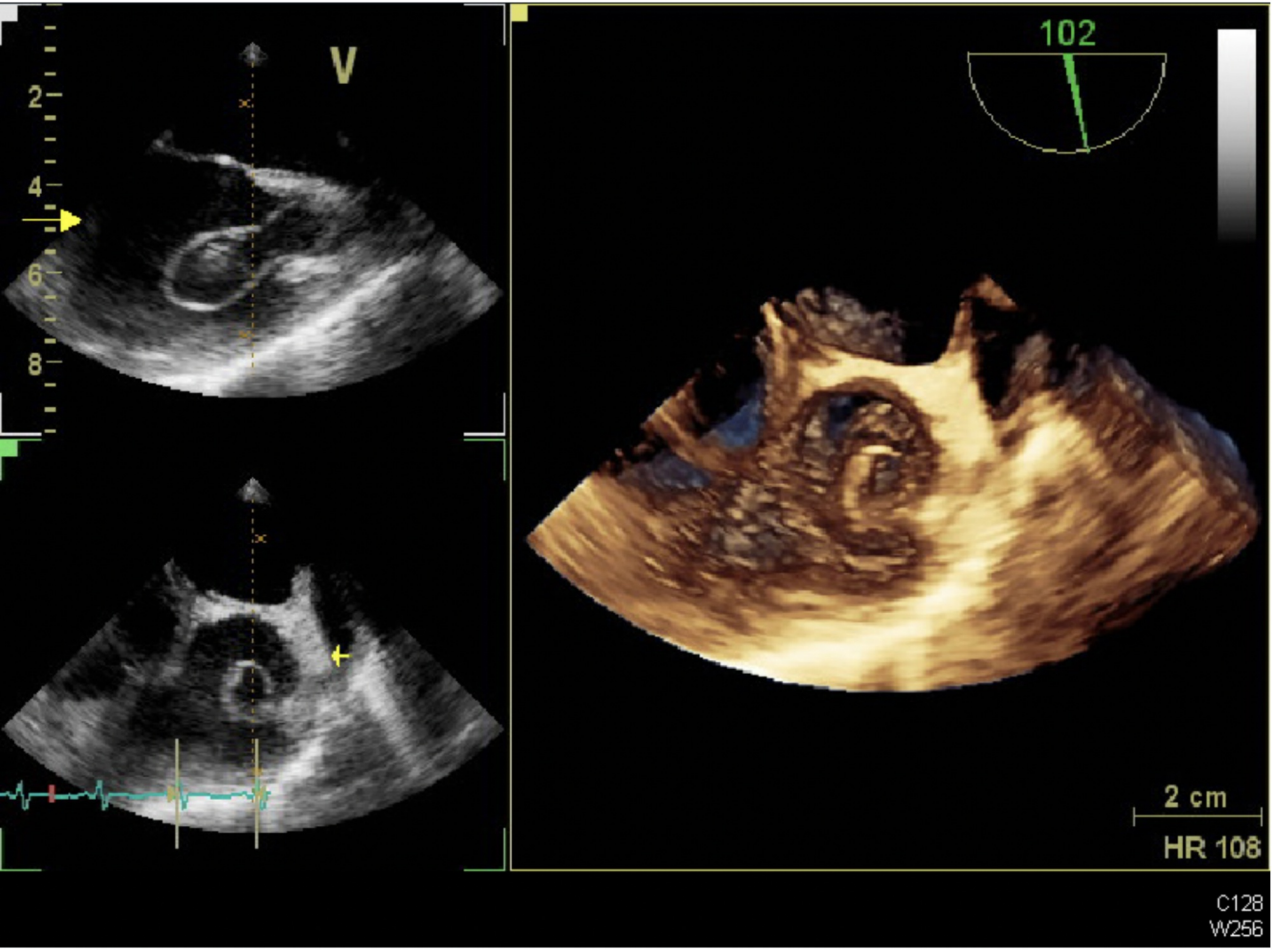
Presenting Transesophageal Echocardiogram (TEE) Imaging TEE images at presentation showing the thrombus-in-transit in the right atrium and at the atrio-caval junction

A follow-up TTE (Figure 1) and TEE (Figure 2) revealed a growing right-sided mobile mass in the right atrium, right ventricle, and pulmonary artery (PA), which was believed to be a large thrombus. 

**Figure 3:**
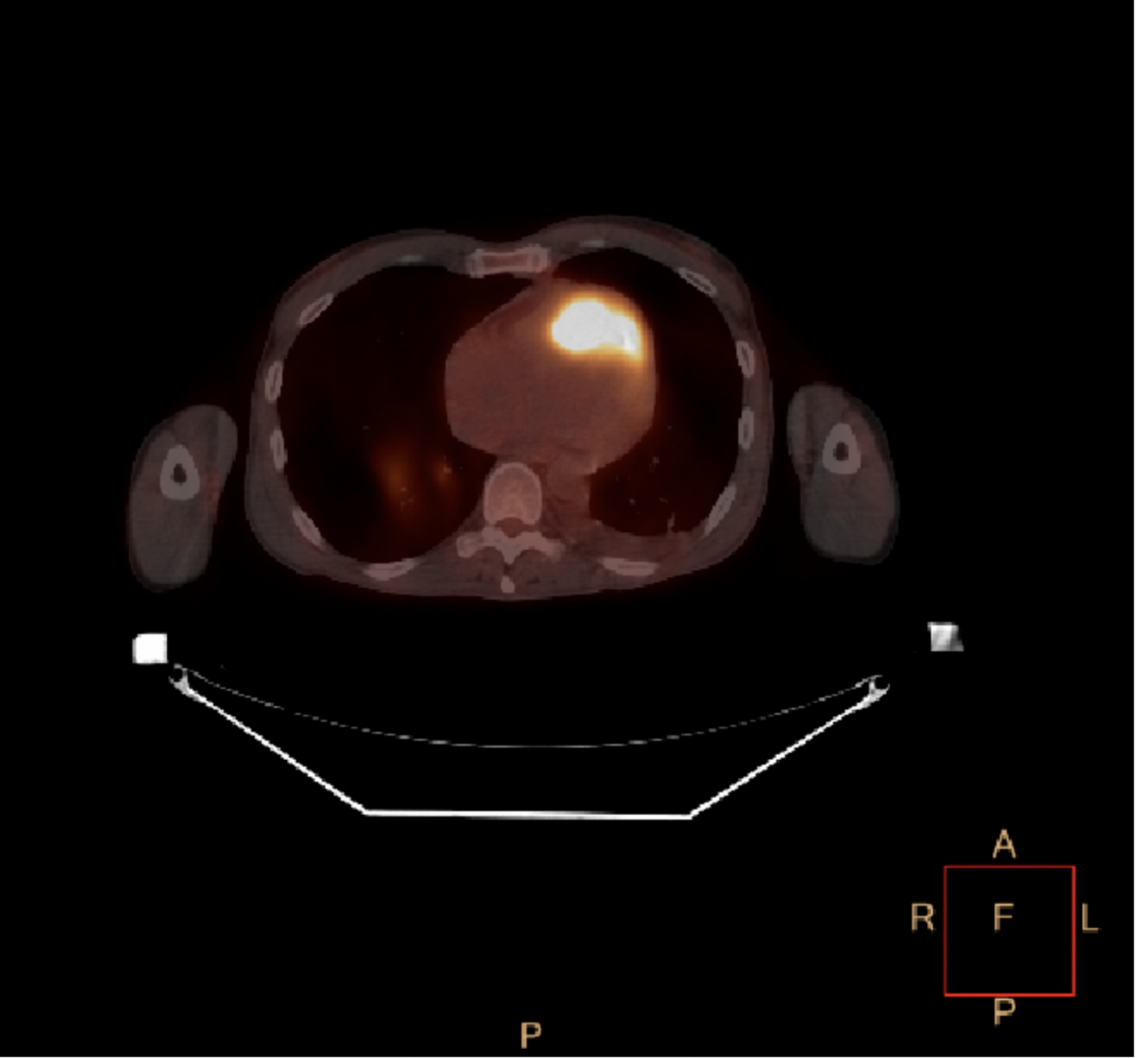
Positron Emission Tomography (PET) Scan PET scan with a hypermetabolic pericardial lesion suggestive of metastatic disease

In view of cardiac tamponade with a rapidly accumulating pericardial effusion on anticoagulation and a presumed larger thrombus burden, interventional radiology and cardiothoracic surgery evaluations were sought. The patient was deemed to be too high-risk for thrombectomy or thrombolytics. At the recommendation of the Oncology Service, in view of the hemorraghic pericardial effusion, a fluorine-18-fluorodeoxyglucose positron emission tomography/computed tomography (18F-FDG PET/CT) scan was performed for further assessment (Figure 3). It revealed that in comparison to a study performed two months prior, the enlarged left subclavicular node appeared more hypermetabolic and had increased in size. The hypermetabolic node in the left posterior hilar region was not apparent on the previous exam. This patient's right infrahilar mass (previously measured 1.6 x 1.2 cm in size) had increased in size and now measured 3.6 x 3.1 cm in size. It was also felt to be more hypermetabolic. A new hypermetabolic left superior pericardial lesion was also noted. 

**Figure 4:**
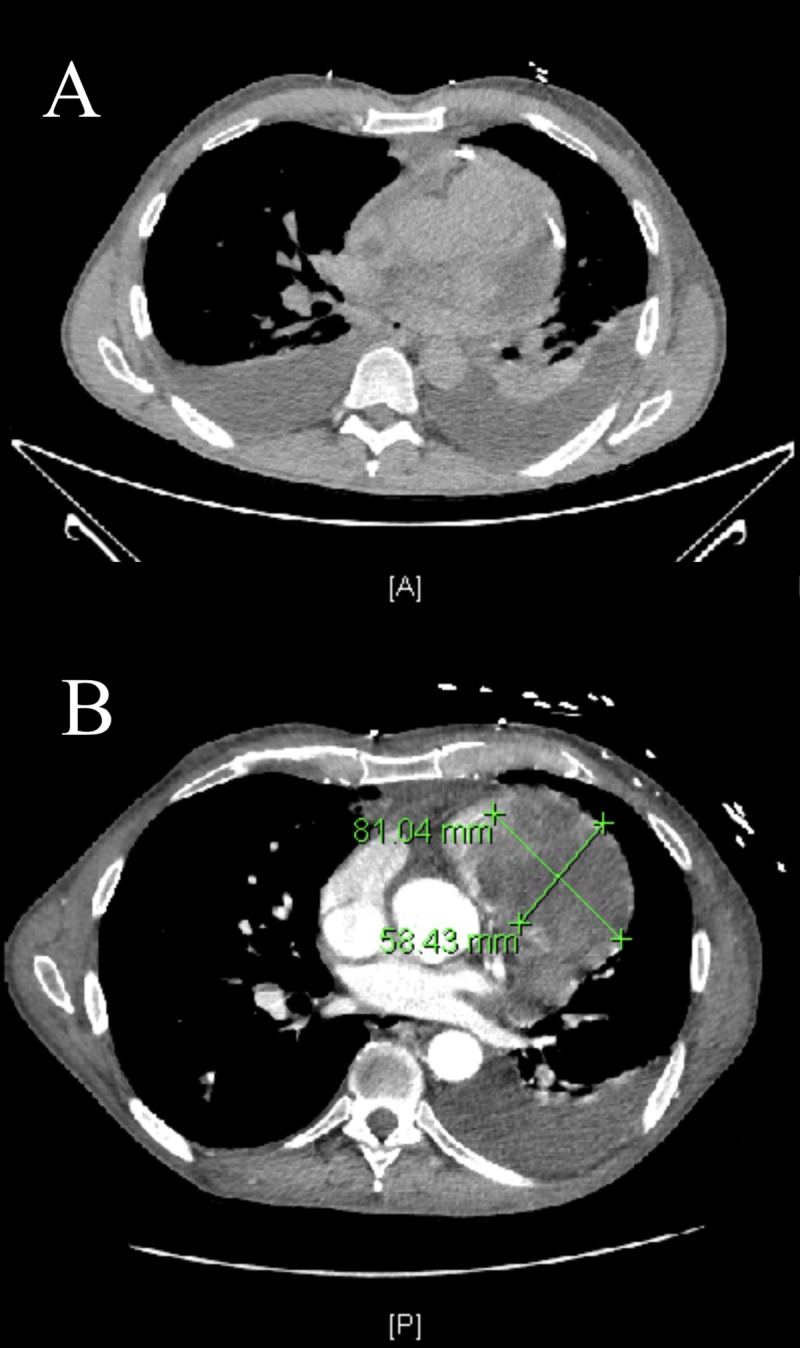
Computed Tomography (CT) Chest Imaging A) Non-contrast CT chest at our institution; B) CT chest with contrast from tertiary care center at a one-week interval showing the development of a large intracardiac mass occupying the right ventricle and the right ventricular outflow tract.

The patient was transferred to a tertiary care center where, after a multidisciplinary assessment, a repeat CT scan of the chest was performed one week after the above-mentioned CT scan (Figure 4A). The interval CT scan of the chest (Figure 4B) revealed a left neck enhancing mass and thrombosis of the left subclavian, brachiocephalic, and atrio-caval junction with collaterals. An acute right upper lung PE was noted. 

**Figure 5:**
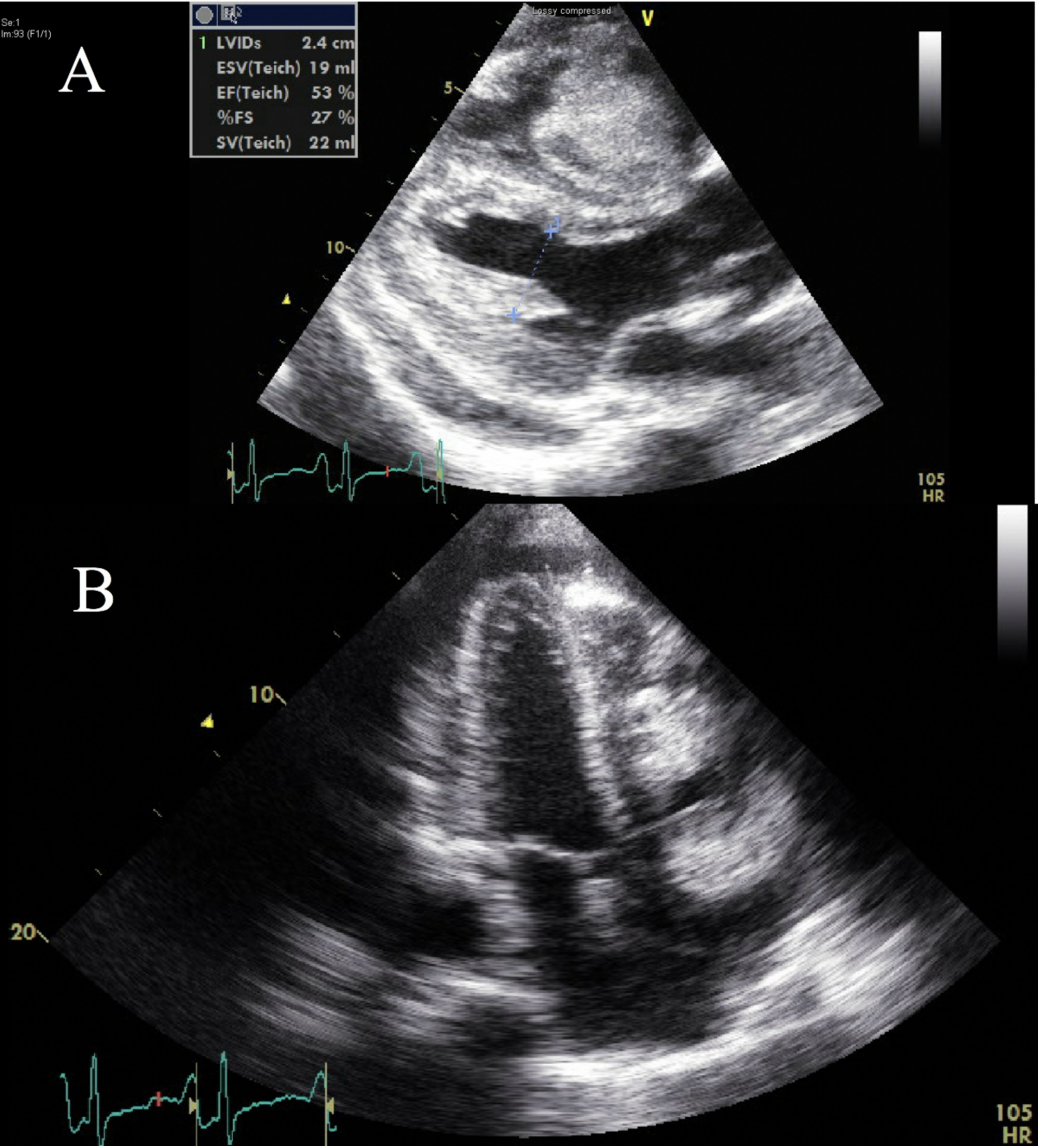
Follow-up Echocardiogram Imaging A) Parasternal long axis and B) apical four-chamber echocardiography views at a six-week interval showing a large mass in the right ventricle.

It also revealed a necrotic, enhancing mass invading the myocardium and right heart measuring 8.1 x 5.8 cm with nodular enhancement of the pericardium with the extension of this mass into the right ventricle outflow tract (RVOT). Hilar and mediastinal lymphadenopathy measuring up to 2.7 cm was also noted. There was no evidence of any venous invasion by the tumor. In view of this, an echocardiogram was obtained at the tertiary care center which revealed a large echodensity in the RVOT and the PA, which appeared to be more echogenic than the thrombus noted elsewhere in the right heart (Figure 5). 

It was concluded that the patient had developed pericardial metastatic disease with erosion into the right ventricle and the RVOT correlating to the new echodense mass that had been noted to develop on the serial echocardiograms. At this time, the patient was discharged on full-dose enoxaparin anticoagulation with plans for nivolumab as an outpatient. Referral to outpatient palliative care was made. 

## Discussion

Cardiac metastatic disease is more commonly found in autopsies than reported in living patients [1-2]. It is far more common than primary cardiac tumors [2-4]. Cardiac metastasis from squamous cell carcinoma of the tongue is highly unusual; it is usually asymptomatic and is usually diagnosed while pursuing non-cardiac symptomatology [5-6]. The mechanism of metastasis to the heart is not well understood. Hematogenous spread through the coronary arteries to the myocardium, direct contiguous extension, and retrograde spread through the lymphatic system have all been postulated as possible mechanisms [7]. In descending order of involvement, tumors typically metastasize to the pericardium, myocardium, epicardium, endocardium, and intracavitary regions [5, 8]. Conduction system involvement is less common. 

Diagnosis of cardiac metastases is often difficult and delayed, as signs and symptoms are nonspecific and variable. Pericardial effusion and cardiac tamponade may often be the first manifestation of cardiac metastasis. Due to its low cost, portability, widespread availability, and lack of ionizing radiation, TTE remains the first-line imaging modality for the evaluation of a patient with a suspected cardiac metastasis. It allows for localization of the mass and evaluation of other cardiac structures and function. TEE provides superior spatial resolution and better visualization of some cardiac masses than TTE, if needed [9-10]. Tissue characterization by means of echocardiography is limited. Our patient challenges this limitation in tissue characterization by TTE and TEE. The intracardiac echodensity was believed to be a large thrombus burden in transit through the right heart. However, this could have been the early stages of tumor transit associated with thrombosis, which these studies failed to differentiate. Also, the hemorrhagic pericardial effusion failed to reveal any malignant cells. The progression of the right heart mass size on the follow-up TTE and TEE at our institution was believed to be consistent with the growing thrombus load, possibly due to the limitation of tissue delineation and concomitant pathologies.

Our patient, with a background history of squamous cell carcinoma of the tongue, posed a significant diagnostic and therapeutic dilemma. Fluorine-18-FDG PET/CT is a useful modality for detecting sites of metastatic disease in cancer patients, but special considerations are required for accurate evaluation of cardiac metastasis [11-12]. Generally, myocardial activity is indeterminate on whole-body FDG PET/CT due to variable background myocardial uptake. However, when focal activity is present relative to background myocardium and biodistribution, as seen in our patient, further imaging is warranted. Combining PET with cardiac CT or cardiac MRI provides a combination of cross-sectional anatomic imaging with metabolic activity. This can be useful in differentiating a benign from a malignant etiology, as well as radiation-induced necrosis from tumor recurrence in a setting of prior surgery [13-15]. In our patient, while the initial TTE and TEE were inconclusive, the PET scan was able to delineate a hypermetabolic pericardial lesion consistent with metastatic disease. This lesion was then noted to have evolved into space-occupying mass lesions in the right ventricle (RV) and RVOT subsequently on both the serial echocardiograms and CT scan of the chest. This combination of the imaging modalities also points towards another rare characteristic of this disease process. The PET scan clearly illustrates that the metastatic lesion was truly pericardial and the intracardiac growth was tumor erosion into the cavity. This is the first reported case of pericardial metastasis from squamous cell carcinoma of the tongue. This unique case highlights the importance of using the complementary nature of various imaging modalities in complex cases. 

Treatments options for patients with cardiac metastatic disease, in general, and especially for cases with aggressive evolution that our patient displayed, are limited. Our patient was deemed not to be a candidate for surgical resection as noted above.

## Conclusions

Cardiac metastasis from squamous cell carcinoma of the tongue is rare in the absence of local recurrence and often has an atypical clinical presentation. Metastatic disease to the heart is more frequently diagnosed post-mortem. While TTE and TEE are typically the first-line imaging modalities for diagnosis in these patients, these modalities often fail to give complete tissue characterization and extent of the disease process. This case exemplifies the importance of serial imaging and integration with PET and CT to enhance antemortem detection of cardiac metastatic disease. We recommend that patients with head and neck malignancies who present with non-specific symptoms should undergo early and serial echocardiographic assessment, and when deemed necessary, a low threshold should be employed for further evaluation with additional cardiac imaging, such as CT, 3D echocardiography, PET, and cardiac magnetic resonance imaging.

## References

[1] Wilkes JD, Fidias P, Vaickus L, Perez RP (1995). Malignancy-related pericardial effusion. 127 cases from the Roswell Park Cancer Institute. Cancer.

[2] Gassman HS, Meadows R Jr, Baker LA (1955). Metastatic tumors of the heart. Am J Med.

[3] Karwinski B, Svendsen E (1989). Trends in cardiac metastasis. APMIS.

[4] Ito T, Ishikawa N, Negishi T, Ohno K (2008). Cardiac metastasis of tongue cancer may cause sudden death. Auris Nasus Larynx.

[5] Bussani R, De-Giorgio F, Abbate A, Silvestri F (2007). Cardiac metastases. J Clin Pathol.

[6] Onwuchekwa J, Banchs J (2012). Early cardiac metastasis from squamous cell carcinoma of the tongue in 2 patients. Tex Heart Inst J.

[7] Werbel GB, Skom JH, Mehlman D, Michaelis LL (1985). Metastatic squamous cell carcinoma to the heart. Unusual cause of angina decubitus and cardiac murmur. Chest.

[8] Reynen K, Köckeritz U, Strasser RH (2004). Metastases to the heart. Ann Oncol.

[9] Butany J, Nair V, Naseemuddin A, Nair GM, Catton C, Yau T (2005). Cardiac tumours: diagnosis and management. Lancet Oncol.

[10] Yusuf SW, Bathina JD, Qureshi S (2012). Cardiac tumors in a tertiary care cancer hospital: clinical features, echocardiographic findings, treatment and outcomes. Heart Int.

[11] Rahbar K, Seifarth H, Schäfers M (2012). Differentiation of malignant and benign cardiac tumors using 18F-FDG PET/CT. J Nucl Med.

[12] Maurer AH, Burshteyn M, Adler LP, Steiner RM (2011). How to differentiate benign versus malignant cardiac and paracardiac 18F FDG uptake at oncologic PET/CT. Radiographics.

[13] Buckley O, Madan R, Kwong R, Rybicki FJ, Hunsaker A (2011). Cardiac masses, part 2: key imaging features for diagnosis and surgical planning. AJR Am J Roentgenol.

[14] Nensa F, Tezgah E, Poeppel TD (2015). Integrated 18F-FDG PET/MR imaging in the assessment of cardiac masses: a pilot study. J Nucl Med.

[15] Motwani M, Kidambi A, Herzog BA, Uddin A, Greenwood JP, Plein S (2013). MR imaging of cardiac tumors and masses: a review of methods and clinical applications. Radiology.

